# Prevalence and determinants of poor sleep quality among diabetic patients in Ethiopia: systematic review

**DOI:** 10.3389/fpubh.2024.1363408

**Published:** 2024-05-14

**Authors:** Hailemicahel Kindie Abate, Abere Woretaw Azagew, Gashaw Adane Nega, Samuel Mersha Birru, Chilot Kassa Mekonnen

**Affiliations:** ^1^Department Medical Nursing, College of Medicine and Health Science, University of Gondar, Gondar, Ethiopia; ^2^Department of Immunology and Molecular Biology, School of Biomedical and Laboratory Science, College of Medicine and Health Science, University of Gondar, Gondar, Ethiopia

**Keywords:** poor sleep quality, diabetes patients, Ethiopia, sleep quality, diabetes sleep

## Abstract

**Background:**

Poor sleep quality can exacerbate many other physiological functions, such as obesity, cardiovascular disease, and high blood pressure. Although primary studies were conducted in Ethiopia, no studies concluded the pooled prevalence of poor sleep quality in Ethiopia. Therefore, this study was conducted to determine the pooled prevalence and its determinants of sleep quality among diabetes in Ethiopia.

**Objective:**

Assess the pooled prevalence and its determinants of sleep quality among diabetes in Ethiopia.

**Methods:**

The studies were searched systematically using international databases from PubMed, Google Scholar, Cochrane Library, Embase, and CINAHL. The quality of the articles searched was assessed using the New Castle Ottawa scale for a cross-sectional study design. Statistical analysis was performed using STATA version 14 and a systematic review was performed using a random effect model method. The Preferred Reporting Item for Systematic Review and Meta-analyses (PRISMA) guideline was followed for reporting results.

**Results:**

From the total of 728 records screened, 8 studies with 2,471 participants who met the inclusion criteria were included in this systematic review. The estimated pooled prevalence of poor sleep quality in Ethiopia was 48.54%.

**Conclusion:**

Almost half of diabetes patients had poor sleep quality. The preparation of brochures on diabetic information and the organization of health education about the negative impact of poor sleep quality on patients are among the best modalities to improve the problem of poor sleep quality.

## Background

Diabetes mellitus (DM) is a syndrome of metabolic disorder due to hyperglycemia as a result of dysfunction of the pancreatic β-cells of the human body (1). Diabetes is one of the leading causes of physiological instability, morbidity, disability, and death of the diabetic population (2).

Sleep is one of the physiological mechanisms of the body that maintains normal body function, tissue stability, and overall health of the body (3). Sleep disorder is one of the main problems for many chronic diseases such as diabetes and many other neurological disorders including migraine headache (4) Poor sleep quality is one of the major problems for diabetic patients compared to those of non-diabetic people. Poor sleep quality can exacerbate many other physiological functions such as obesity, cardiovascular diseases, and high blood pressure (5, 6). Sleep deprivation in diabetes patients leads to long- and short-term effects that include increased anxiety and stress, reduced quality of life, major mental disorders, and other chronic medical conditions (7). Poor sleep quality prominently affects diabetic patients by increasing insulin resistance, long-term diabetes complications, and failure of major organs (8). Insufficient sleep is associated with many other negative health outcomes and increases the mortality of diabetes patients (9).

Based on the report of the International Diabetes Federation (IDF) for 2019, it was estimated that 9.3% (463 million) of the world population was diabetic cases (10). A systematic review in Ethiopia showed that the prevalence of diabetes ranges from 2 to 6.5% (11). Along with the increase in the prevalence of diabetics, the quality of sleep of individuals has been rising in the past decades (12). Evidence showed that long-lasting sleep deprivation was estimated to affect 7.5–20% total population (13). This problem is neglected until patients come to health institutions with other comorbidities (14). Various studies around the world have shown that many people who are faced with chronic diseases are faced with chronic poor sleep quality (15–17). This chronic sleep quality can lead to a poor prognosis of the disease, impaired exercise tolerance, decreased self-care behavior, and decreased total quality of life (18, 19).

Poor sleep quality in diabetics was associated with many sociodemographic variables, such as being female, low wealth status, longer duration of the disease, poor body glucose control, and the presence of other comorbidities of chronic disease (20, 21).

In Ethiopia, different studies were conducted to estimate the prevalence of sleep quality and associated factors among diabetic patients. The estimated prevalence of poor sleep quality in Ethiopia ranges from 31.97% (22) to 80.7% (23) which was conducted in the Amhara regional state of Amhara in Ethiopia. The findings of these different studies showed that there were significant variations in the prevalence of poor sleep quality among adult patients with diabetes in Ethiopia.

Regarding the researchers’ search, there was no systematic review conducted in Ethiopia related to this review. The findings of this study will be used as feedback to healthcare care managers to formulate or revise policies to improve poor sleep quality. Therefore, this study was conducted to determine the pooled prevalence and its determinants of sleep quality among diabetes in Ethiopia.

## Methods

This study was guided by the Newcastle-Ottawa Scale for cross-sectional studies for systematic review (24, 25) and it was carried out based on the preferred reporting items for systematic review (26). This systematic review title and its protocol were registered in the PROSPERO online database with the registration number CRD42023444939.

### Search strategies

International databases (PubMed, Scopus, Web of Science, and Cochran Library) and search engines (Google and Google Scholar) were used to locate research articles on the prevalence of poor sleep quality among diabetic patients in Ethiopia. The search string was developed using the Boolean operators “AND” and “OR” with keywords extracted from the Medical Subject Headings (MeSH) database. The search strategy was based on the research question of this review and used CoCoPop (Co = Condition, Co = Context, Pop = Population).

The article location strategy was through “poor sleep quality” OR “sleep quality” AND “diabetes” AND “Ethiopia”.

This search strategy aimed primarily to trace all primary studies reviewed (published) and unpublished. Sources of information range from electronic databases to direct contact with the principal investigator if mandatory. The first search through Pub Med, Cochran Library, Scopus, Web of Science, Google, and Google Scholar was carried out in March 2023. The final update search was carried out from July 6, 2023, to September 29, 2023. The publication date was used as a filter mechanism in which articles published from May 2017 to August 29/2023 in this systematic review and meta-analysis study were used to generate the most recent evidence for the scientific community.

### Eligibility criteria

#### Study inclusion and exclusion criteria

Quantitative studies that reported the prevalence of overall poor sleep quality among diabetic patients, master’s thesis, and dissertations were included in the study, while qualitative study design, single case study research reports, not fully accessed articles, and poor methodological quality were excluded from the analysis.

#### Study selection and results

After a comprehensive search, all located citations were selected and exported to Endnote Citation Manager software version X7. Subsequently, irrelevant and duplicate articles were removed. Then, two independent researchers (CKM and AWA) screened each particular article by far for its title, abstract, and full text and cross-checked it against the inclusion criteria. The other research team (HKA, MCA, and AFZ) verified the articles selected with the full text for details under the already defined criteria to take them to the final review process. Any kind of disagreement between the research team while including and excluding articles will be managed by predefined criteria. The result of the search for the further selection and inclusion process of articles in this review was carried out by the PRISMA guidelines for systematic review and meta-analysis 2020.

### Quality appraisal of included studies

Articles searched in the database were collected and duplicate articles were manually removed using EndNote (version 7). The modified version of the Newcastle-Ottawa Scale is used as a quality assessment tool for cross-sectional studies to evaluate the quality of the included studies (25). This critical appraisal tool consists of three items that assess the selection of study subjects (which consists of four different questions that account for a maximum of 5 points), the comparability of the study (which has two different questions that account for 3 points for each), and the outcome of the study (which contains one question and a maximum 1 point for each) (Supplementary Table S1). After critical evaluation, the reviewers decided to include or exclude screened articles based on the overall quality of the evaluation score of 9.

Two independent reviewers evaluated the methodological quality of each article before inclusion in the review using nine points from the three sections of the tool. The quality of each study was rated using three categorical algorithms. A scores ≥7 was considered “good quality” of the study, a score 4–6 was considered “fair quality” of the studies, and studies with quality ≤3 was “poor quality.” Studies with a final quality score of 4 points and above were included in the final review (25).

In this sense, there had to be a study (27) with poor quality categories to exclude the article from the review. Any sort of disagreement between the involved reviewers was resolved through the discussion of the reviewers. Furthermore, if disagreement develops, the fourth reviewer is indicated to supervise the source of doubt and reach a consensus. The exclusion of articles was presented with countable reasons that could be consistent with the predefined criteria. The result of the search for the further selection and inclusion process of articles in this review was carried out in agreement with the PRISMA guidelines for systematic review and meta-analysis 2020.

Data were independently extracted by four authors using a 2014 Joanna Briggs Institute Reviewers Manual (JBI) (28).

The tool includes authors; study year, study design, sample size, prevalence, and risk of bias assessment score were included in the extraction. Data were extracted by two independent reviewers and any inconsistent data was cross-checked (Supplementary Table S2). Disagreement between reviewers was resolved through discussion.

### Outcome measurements

The primary results of this meta-analysis and systematic review were the pooled prevalence of poor sleep quality among diabetes patients elsewhere in Ethiopia.

### Data synthesis and analysis

The outcome of the included primary studies was presented narratively and expanded with additional material in text, tables, and figures, where necessary. All necessary and relevant information for each article was extracted using a Microsoft Excel spreadsheet and exported to STATA Version 11 for further analysis. The random effect model was used to estimate the size of the pooled effect of patients with poor sleep quality diabetes due to the presence of heterogeneity (29). The size of the pooled effect with a 95% confidence interval was presented by a forest plot and used to visualize the presence of heterogeneity graphically. For the possible difference in the primary study, we explored subgroup analysis and meta-regression subsequently using publication year, study design, study setting, sampling methods, sample size, sex, and region. Additionally, publication bias was evaluated through visual inspection of the funnel plot, Begg-Mazudar rank correlation test, and Egger test to see the funnel plot’s asymmetry (30). The influence of individual articles on the estimate of the overall pooled effect size or the prevalence of poor sleep quality among diabetes was assessed using sensitivity analysis. The forest plot with 95% CI was used to present the overall pooled prevalence, as well as the pooled subgroup prevalence of poor sleep quality among diabetes in Ethiopia. The logarithmic odds ratio was used to determine the associated factors of poor sleep quality among patients with diabetes in Ethiopia.

## Result

### The systematic review

#### Article selection and outcomes

**Figure 1:**
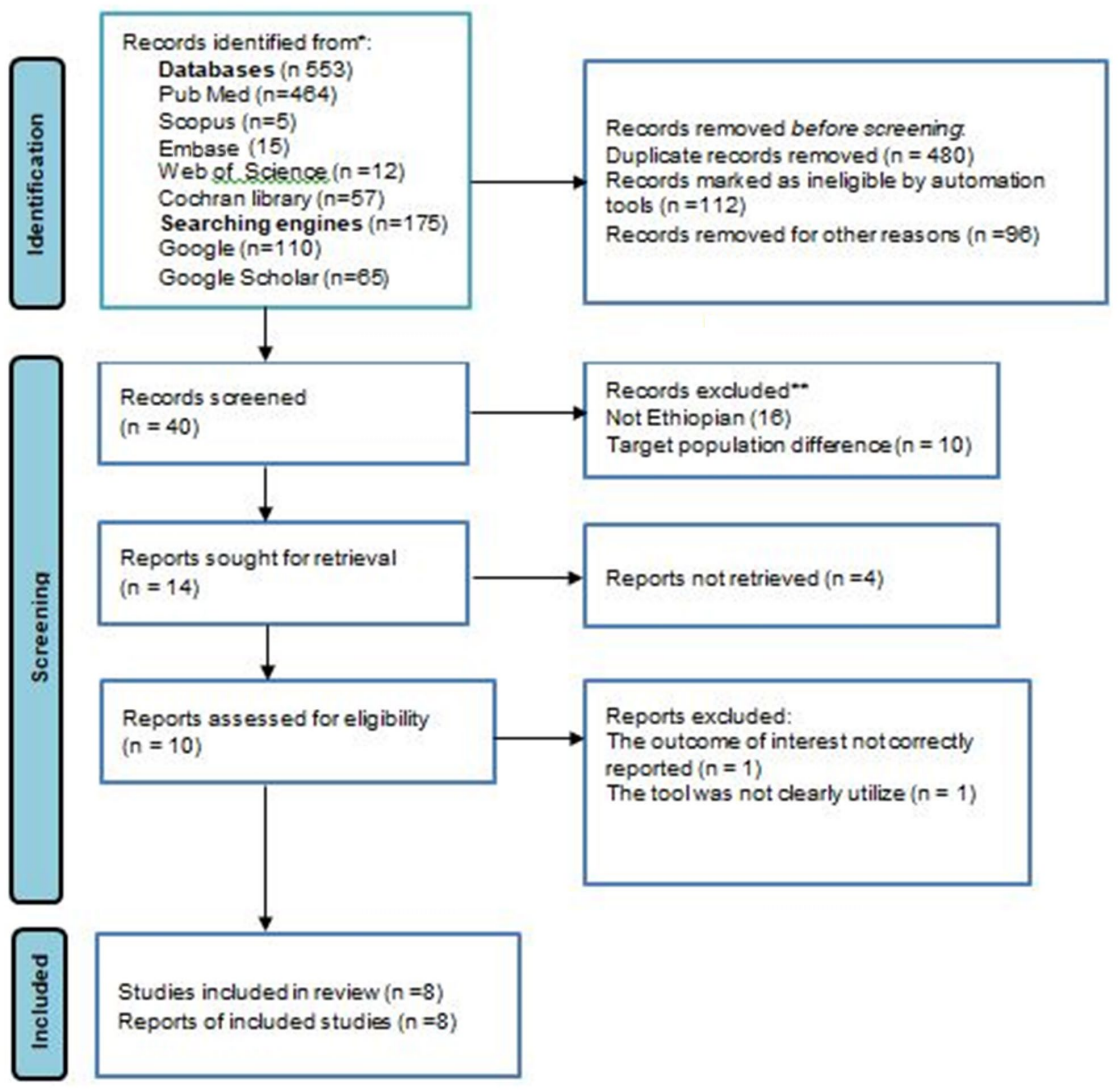
PRISMA flow diagram of the included studies.

In this systematic review and meta-analysis study, a total of 728 articles related to the prevalence of nonadherence to exercise in Ethiopia were identified using electronic databases and search engine websites. Among the overall articles found, 480 were removed because they were irrelevant and duplicated and the other 112 and 96 were removed because they were not eligible (study design and title difference) due to automation tools and other reasons, respectively. The remaining 40 articles were eligible for selection. Of these screened, 26 articles were excluded due to the study region not being conducted in Ethiopia and not similar to the target population. On further screening, 14 articles were sought for retrieval and 4 were not retrieved for one or the other reason. Furthermore, 10 research articles were evaluated for eligibility to be included in the review process, but with the outcome of interest and the ambiguity of the measurement tool, a total of 2 articles were excluded. Finally, eight original research articles were incorporated for systematic review and meta-analysis (Figure 1).

### The methodological quality assessment of the included studies

A total of 8 articles have been assessed for methodological quality using the Newcastle-Ottawa scale of 9 points for the quality assessment tool for cross-sectional studies quality assessment tool (31). The outcome of the quality evaluation ranged from moderate to high methodological quality, in which two studies scored 9 points (19, 23), three studies scored (22, 27, 32) and the other two studies scored (33–35) (Supplementary Table S1).

### Description of included studies

All research articles included in this systematic review were carried out by cross-sectional study design and published between January 2013 and September 29/2023. Eight published studies with 2,471 participants were included to determine the combined prevalence of poor sleep quality among diabetes patients. All articles were carried out with a cross-sectional study design with the lowest and highest prevalence of poor sleep quality in the state of Ethiopia 31.97% (22) and (80.7%) (32), respectively. In the same way, the smallest and largest sample sizes were from the Amhara region 66 (32) and 614 (33), respectively. This review includes six studies from the Amhara region (19, 22, 23, 32, 33), and two studies from the Oromia (27, 34) region of Ethiopia (Table 1).

**Table 1:** Summary of the prevalence of poor sleep quality among diabetes using eight studies included in the systematic review and meta-analysis.

Author/year of publication	Year	Region	Sample method	Sample size	Prevalence (%)	Response rate (%)	Quality
Debalke et al. (27)	2020	Oromia	Consecutive sampling	253	45.5	100	9
Bayush el al. (33)	2022	Amhara	simple random sampling	614	45.9	97	9
Edmealem et al. (19)	2021	Amhara	Simple random sampling	344	36	100	8
Jemere et al. (23)	2023	Amhara	Systematic random sampling	99	55.6	100	7
Mersha et al. (32)	2022	Amhara	Consecutive sampling	63	80.7	100	8
Wonde et al. (34)	2022	Oromia	Consecutive sampling	204	42.2	95.2	6
Worku et al. (22)	2023	Amhara	Systematic random sampling	319	31.97	100	7
Zewdu et al. (35)	2022	Amhara	Systematic random sampling	575	50.7	100	8

### The pooled prevalence of poor sleep quality of diabetes in Ethiopia

**Figure 2:**
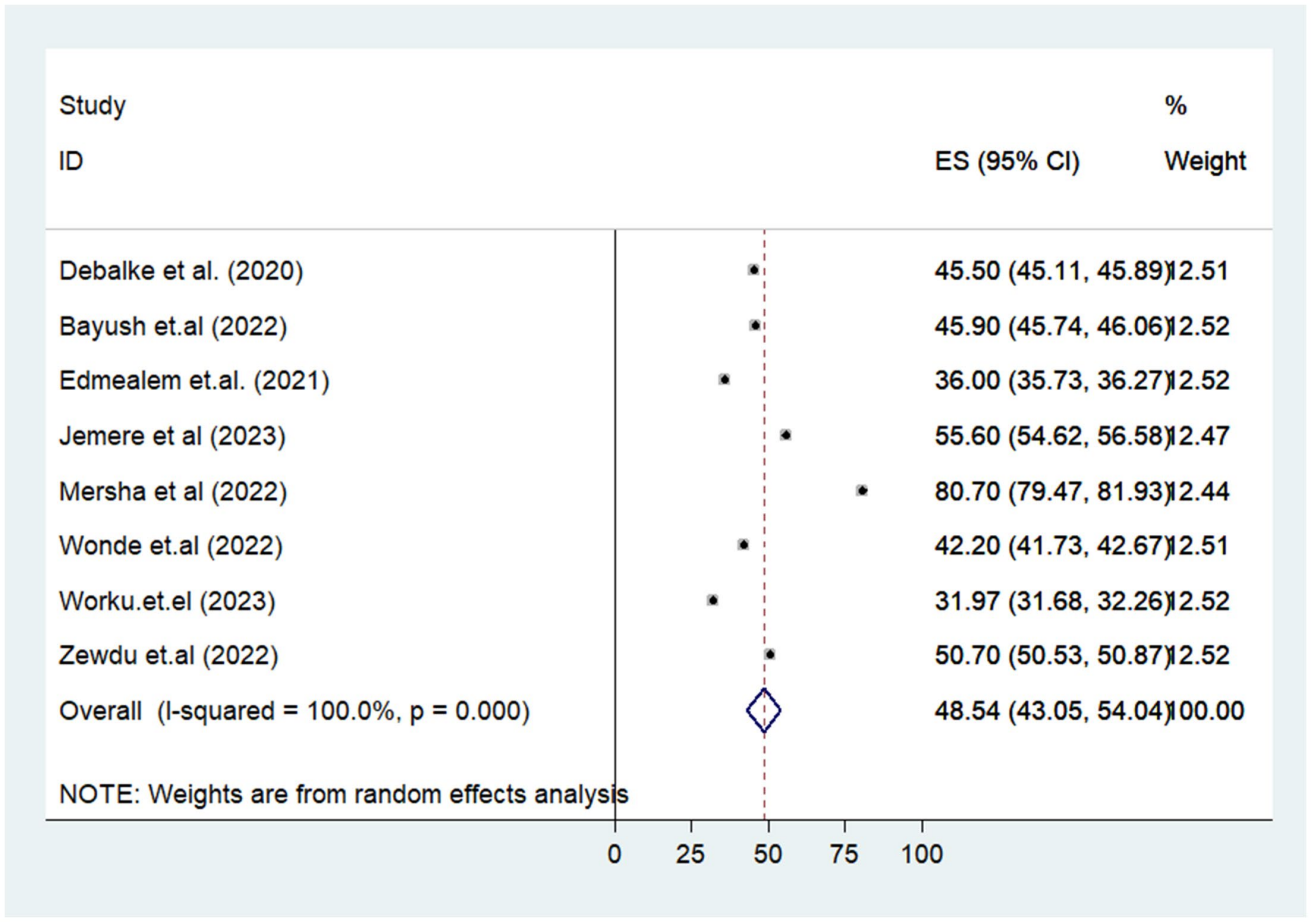
Forest plot of the prevalence of poor sleep quality among diabetes patients in Ethiopia.

The prevalence of poor sleep quality ranges from 31.97% (22) to 80.70% (32) in the Amhara regional state of Ethiopia. The pooled prevalence of poor sleep quality in patients with diabetes was 48.54% with 95% CI (43.55–04.04) based on the analysis of the DerSimonian-Laird random-effects model. The lowest and highest pooled prevalence of poor sleep quality among diabetes patients was found in the Amhara region in Ethiopia at 31.97% (95% CI (31.68–32.26)) and 80.7% (95% CI (79.47–81.93)), respectively (Figure 2).

### Assessment of heterogeneity

In this systematic review, a subgroup analysis was performed to assess heterogeneity, so that the *p*-value and I^2^ statistics were used to assess heterogeneity between studies.

The source of heterogeneity was using the region, study years, and sampling techniques to identify the reason for the variation between studies, but none of them is the source of heterogeneity (Table 2).

**Table 2:** Subgroup analysis of pooled prevalence poor sleep quality among diabetes patients in Ethiopia, 2023.

Subgroups		Number of studies	Pooled prevalence	*p*-value	*Ι*^2^ (%)
Sampling technique	Consecutive sampling	3	56.11 (42.86–69.37)	<0.001	99.9
	Simple random sampling	5	40.95 (31.25–50.65)	<0.001	100
	Systematic random sampling		46.09 (31.51–60.66)	<0.001	100
Study period	<2020	4	42.25 (35.13–49.37)	<0.001	99.9
	≥2020	4	54.80 (49.50–60.09)	<0.001	99.9
Region	Amhara	6	48.54 (43.05–54.04)	*p* < 001	100
	Oromia	2	43.85 (40.62–47.09)	*p* < 001	99.1

### Meta-regression

Furthermore, subgroup analysis and univariate meta-regression with sample size and publication year were performed for possible heterogeneity. The result of the analysis indicates that none of them significantly affected the heterogeneity between studies (Table 3).

**Table 3:** Meta-regression analysis of studies of poor sleep quality among diabetes patients in Ethiopia, 2023.

Heterogeneity	Coef.	Std. Err.	*t*	*p* > *t*	[95% Conf.]
Publication year	−0.025	0.072	−0.35	0.744	−0.212604–0.1622628
Sample size	0.001	0.0003	0.82	0.451	−0.00058–0.00112

### Publication bias

**Figure 3:**
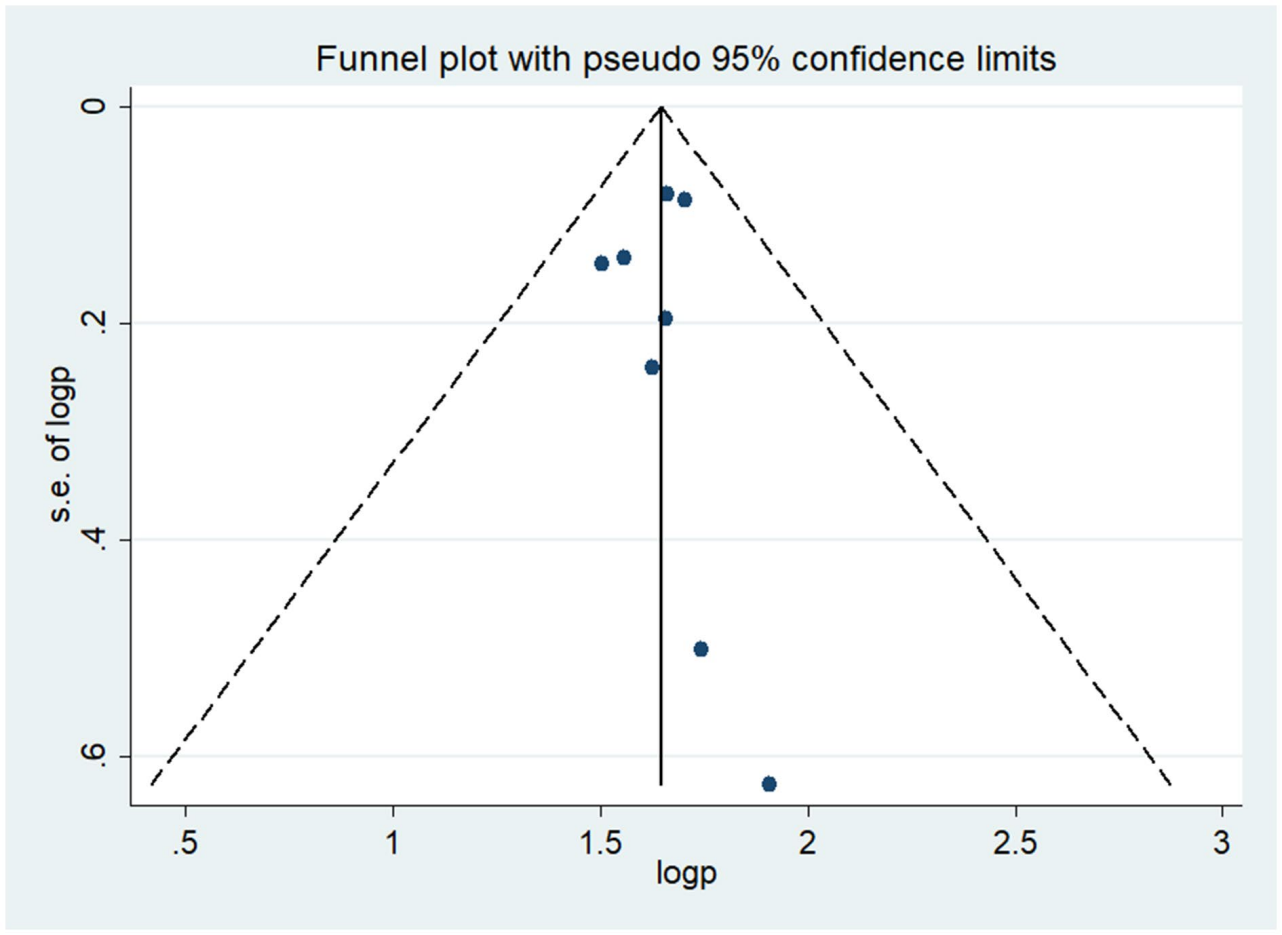
Funnel of poor sleep quality among diabetes in Ethiopia.

The funnel plot was done to show to check the publication bias. This study has no publication bias, as it is symmetrically distributed. Furthermore, statistically, the Begg and Egger test was performed with *p*-value = 0.42, which showed that there was no publication bias (Figure 3).

### Sensitivity analysis

**Figure 4:**
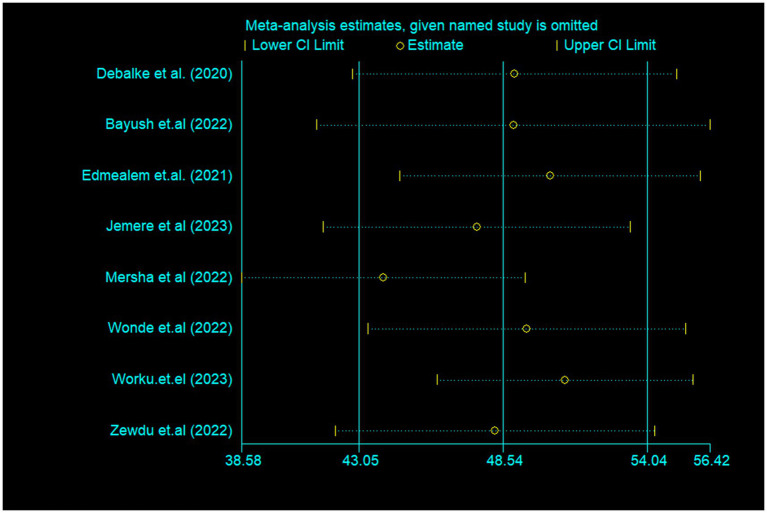
Sensitivity analysis that indicates poor sleep quality among diabetes in Ethiopia.

A sensitivity analysis was performed after observing lower and higher values in the review to show the effect of one study on the overall summary effect. However, the result of the analysis of the sensitivity test using the random effects model indicated that there was no single effect on the overall estimate (Figure 4).

## Discussion

In this systematic review and meta-analysis, the pooled prevalence of poor sleep quality among diabetes patients was 48.54% with 95% CI (43.05–54.04). Although there was no similar systematic review or meta-analysis evidence, related studies were conducted across the world. The finding of this study was supported by a meta-analysis conducted in (Asia, Europe, America, and Oceania countries) (36). Another related meta-analysis study conducted in Italy showed that the quantity and quality of sleep were poor among diabetic patients (37). The finding of this study was also supported by a systematic review and meta-analysis conducted in the Asia population (38). The possible justification might be that people who are diabetics might encounter diseases such as nocturia, nocturnal hypoglycemia, restless leg syndrome, peripheral neuropathy, and impaired breathing during sleep. These mentioned factors cause fragmented sleep and poor quality of sleep (39).

The findings of this study were lower than those of studies conducted in Jordan (81%) (40), in the USA (55%) (41), Ghana (64%) (42). The possible justification could be that poor sleep quality in developed countries might be high due to the occurrence of diabetic complications increasing as the duration of the disease is longer. Furthermore, diabetic patients with poor glycemic control developed nocturia that led to frequent awakenings resulting in poor sleep quality of diabetic patients (43).

The finding of this study was consistent with the studies conducted in China (47.1%) (44), Thailand (48.4%) (45), and Myanmar (48.4%) (45). The possible justification could be that diabetic patients may have similar physiological complications such as nocturia, nocturnal hypoglycemia, restless leg syndrome, sedentary breathing, and diabetic and heart failure problems. All of the above physiological complications can influence the normal sleep hours of diabetic patients (39).

The finding of this study was higher than that of the study conducted in Malaysia (32%) (46).

The possible justification could be because so many behavioral factors could be increased in developing countries, which could increase the quality of sleep quality of patients (47).

### Limitations of the study

The studies included in this review were cross-sectional as a result; the outcome variable could be affected by other confounding variables. Furthermore, some regions of the country were not included because a lack of research may lead to an underestimate of this review.

## Conclusions and recommendations

This systematic review and meta-analysis revealed that nearly half of diabetic patients had poor sleep quality compared to most previous studies conducted elsewhere in the world. This national evidence would be helpful for cross-country comparisons of the proportion of poor sleep quality among diabetes patients. It may be useful for healthcare policymakers to emphasize the overall quality of service by incorporating components of lifestyle modification, particularly for patients with substance use. The preparation of brochures on diabetic information and the organization of health education about the negative impact of poor sleep quality on patients are among the best modalities to improve the problem of poor sleep quality. The authors recommended that the coming researcher’s better conduct on continent-wide sleep quality of diabetic patients.

## Data availability statement

The original contributions presented in the study are included in the article/Supplementary material, further inquiries can be directed to the corresponding author.

## Author contributions

HA: Conceptualization, Data curation, Formal analysis, Funding acquisition, Investigation, Methodology, Project administration, Resources, Software, Supervision, Validation, Visualization, Writing – original draft, Writing – review & editing. AA: Conceptualization, Data curation, Formal analysis, Funding acquisition, Investigation, Methodology, Project administration, Resources, Software, Supervision, Validation, Visualization, Writing – original draft, Writing – review & editing. GN: Conceptualization, Data curation, Formal analysis, Funding acquisition, Investigation, Methodology, Project administration, Resources, Software, Supervision, Validation, Visualization, Writing – original draft, Writing – review & editing. SB: Conceptualization, Data curation, Formal analysis, Funding acquisition, Investigation, Methodology, Project administration, Resources, Software, Supervision, Validation, Visualization, Writing – original draft, Writing – review & editing. CM: Conceptualization, Data curation, Formal analysis, Funding acquisition, Investigation, Methodology, Project administration, Resources, Software, Supervision, Validation, Visualization, Writing – original draft, Writing – review & editing.

## Supplementary material

SUPPLEMENTARY TABLE S1 Quality appraisal and data extraction of included studies.

SUPPLEMENTARY TABLE S2 Methodological quality assessment of included studies.
