# Supplementary material for: Prevalence and determinants of poor sleep quality among diabetic patients in Ethiopia: systematic review
Source: Front Public Health. 2024 May 14;12:1363408. doi: 10.3389/fpubh.2024.1363408 (PMC11130494; doi:10.3389/fpubh.2024.1363408)
Supplement: SUPPLEMENTARY TABLE S1 — Quality appraisal and data extraction of included studies. [file Table_1.doc]

Table S2: The methodological quality of included studies using the modified version of Newcastle-Ottawa Scale for cross-sectional studies

| Included studies | Selection criteria point | | | | Comparability criteria point | Outcome criteria point | | Total /9 |
| --- | --- | --- | --- | --- | --- | --- | --- | --- |
|  | Q1s | Q2s | Q3s | Q4s | Q1c | Q1O | Q2O |
| Debalke et al. | 1 | 1 | 1 | 2 | 2 | 1 | 1 | 8 |
| Bayush et.al | 1 | 0 | 1 | 2 | 1 | 1 | 1 | 7 |
| Edmealem et.al. | 1 | 1 | 1 | 1 | 2 | 1 | 1 | 9 |
| Jemere et al | 1 | 1 | 1 | 2 | 2 | 1 | 1 | 9 |
| Mersha et al | 1 | 1 | 1 | 0 | 2 | 1 | 1 | 8 |
| Wonde et.al | 1 | 0 | 1 | 0 | 2 | 0 | 1 | 7 |
| Worku.et.el | 1 | 1 | 0 | 2 | 2 | 1 | 1 | 8 |
| Zewdu et.al | 1 | 1 | 1 | 1 | 2 | 1 | 0 | 7 |

Q1s: Representativeness of the cases, Q2s: Sample size: Q3s: Non-Response rate Q4s: Ascertainment of the screening/surveillance tool, Q1c: potential confounders were investigated, Q1O: Assessment of the outcome, Q2O: is statistical test used to analyze the data
