# Supplementary material for: Prevalence and determinants of poor sleep quality among diabetic patients in Ethiopia: systematic review
Source: Front Public Health. 2024 May 14;12:1363408. doi: 10.3389/fpubh.2024.1363408 (PMC11130494; doi:10.3389/fpubh.2024.1363408)
Supplement: SUPPLEMENTARY TABLE S2 — Methodological quality assessment of included studies. [file Table_2.doc]

Supplementary file Table 1: Summary of the prevalence of poor sleep quality among eight studies of included in the systematic review and meta-analysis

| Author/year of publication |  | Region | Sample method | Sample size | Prevalence  (%) | Response rate (%) | Quality |
| --- | --- | --- | --- | --- | --- | --- | --- |
| Debalke et al. | 2020 | Oromia | Consecutive sampling | 253 | 45.5 | 100 | 9 |
| Bayush el. al. | 2022 | Amhara | simple random sampling | 614 | 45.9 | 97 | 9 |
| Edmealem et al. | 2021 | Amhara | Simple random sampling | 344 | 36 | 100 | 8 |
| Jemere et. Al. | 2023 | Amhara | Systematic random sampling | 99 | 55.6 | 100 | 7 |
| Mersha et.al. | 2022 | Amhara | Consecutive sampling | 63 | 80.7 | 100 | 8 |
| Wonde et.al | 2022 | Oromia | Consecutive sampling | 204 | 42.2 | 95.2 | 6 |
| Worku | 2023 | Amhara | Systematic random sampling | 319 | 31.97 | 100 |  |
| Zewdu et.al | 2022 | Amhara | Systematic random sampling | 575 | 50.7 | 1oo | 8 |

Table 2: Subgroup analysis of the pooled prevalence physical exercise non-adherence among type 2 diabetes patients in Ethiopia.

| subgroups | | number of studies | Pooled prevalence | heterogeneity statistics | p-value | I^2^ | Tau  squared |
| --- | --- | --- | --- | --- | --- | --- | --- |
| Sampling technique | Multistage | 2 | 67.40(55.24-79.55) | 150000 | < 0.001 | 100% | 76. 87 |
|  | Systematic random sampling | 5 | 43.86 (21.63-66.08) | 20418 | < 0.001 | 100% | 642.69 |
| Study period | < 2020 | 3 | 51.47 (36.67-66.27) | 150000 | < 0.001 | 100 % | 1300 |
|  | ≥ 2020 | 4 | 50.58 (34.59-66.57) | 99948.91 | < 0.001 | 100 % | 227.98 |
| Study setting | Hospital | 5 | 43.86(21.64-66.08) | 150000 | P<001 | 100% | 642.69 |
|  | Community | 2 | 67.40(55.25-79.55) | 20418.30 | P<001 | 100% | 76.87 |

NB: *others; systematic and stratified sampling

Table 3: Meta-regression analysis of studies physical exercise non-adherence Ethiopia, 2023.

| Heterogeneity | Coefficients | Std. Err. | p-value |
| --- | --- | --- | --- |
| Publication year | .086862 | .0355569 | 0.071 |
| Sample size | -.0000518 | .0002789 | 0.862 |
